# Seroprevalence of *Toxoplasma gondii* infection in arthritis patients in eastern China

**DOI:** 10.1186/s40249-017-0367-2

**Published:** 2017-10-25

**Authors:** Ai-Ling Tian, Yuan-Lin Gu, Na Zhou, Wei Cong, Guang-Xing Li, Hany M. Elsheikha, Xing-Quan Zhu

**Affiliations:** 1 State Key Laboratory of Veterinary Etiological Biology, Key Laboratory of Veterinary Parasitology of Gansu Province, Lanzhou Veterinary Research Institute, Chinese Academy of Agricultural Sciences, Lanzhou, Gansu Province 730046 People’s Republic of China; 2 Weihaiwei People’s Hospital, Weihai, Shandong Province 264200 People’s Republic of China; 3 Affilliated Hospital of Medical College, Qingdao University, Qingdao, Shandong Province 266000 People’s Republic of China; 4 School of Basic Medicine, Dali University, Dali, Yunnan Province 671000 People’s Republic of China; 5 Faculty of Medicine and Health Sciences, School of Veterinary Medicine and Science, University of Nottingham, Sutton Bonington Campus, Loughborough, LE12 5RD UK

**Keywords:** *Toxoplasma gondii*, Autoimmunity, Arthritis, Seroprevalence, Risk factors

## Abstract

**Background:**

There is accumulating evidence for an increased susceptibility to infection in patients with arthritis. We sought to understand the epidemiology of *Toxoplasma gondii* infection in arthritis patients in eastern China, given the paucity of data on the magnitude of *T. gondii* infection in these patients.

**Methods:**

Seroprevalence of *T. gondii* infection was assessed by enzyme-linked immunosorbent assay using a crude antigen of the parasite in 820 arthritic patients, and an equal number of healthy controls, from Qingdao and Weihai cities, eastern China. Sociodemographic, clinical and lifestyle information on the study participants were also obtained.

**Results:**

The prevalence of anti-*T. gondii* IgG was significantly higher in arthritic patients (18.8%) compared with 12% in healthy controls (*P* < 0.001). Twelve patients with arthritis had anti-*T. gondii* IgM antibodies – comparable with 10 control patients (1.5% vs 1.2%). Demographic factors did not significantly influence these seroprevalence frequencies. The highest *T. gondii* infection seropositivity rate was detected in patients with rheumatoid arthritis (24.8%), followed by reactive arthritis (23.8%), osteoarthritis (19%), infectious arthritis (18.4%) and gouty arthritis (14.8%). Seroprevalence rates of rheumatoid arthritis and reactive arthritis were significantly higher when compared with controls (*P* < 0.001 and *P* = 0.002, respectively). A significant association was detected between *T. gondii* infection and cats being present in the home in arthritic patients (odds ratio [*OR*], 1.68; 95% confidence interval [*CI*]: 1.24 – 2.28; *P* = 0.001).

**Conclusions:**

These findings are consistent with and extend previous results, providing further evidence to support a link between contact with cats and an increased risk of *T. gondii* infection. Our study is also the first to confirm an association between *T. gondii* infection and arthritis patients in China. Implications for better prevention and control of *T. gondii* infection in arthritis patients are discussed.

**Trial registration:**

This is an epidemiological survey, therefore trial registration was not required.

**Electronic supplementary material:**

The online version of this article (10.1186/s40249-017-0367-2) contains supplementary material, which is available to authorized users.

## Multilingual abstracts

Please see Additional file 1 for translation of the abtract into the five official working languages of the United Nations.

## Background

Toxoplasmosis is a parasitic disease caused by infection with the obligate intracellular apicomplexan protozoan *Toxoplasma gondii*. This parasite is able to infect all warm-blooded animals and chronically infects approximately one-third of the world’s human population [1]. In immunocompetent individuals, *T. gondii* infection induces no apparent morbidity. In immunocompromised individuals however, or patients undergoing immunosuppressive treatments or during pregnancy, infection with *T. gondii* can cause serious clinical consequences and even death [2–5]. Many of the clinical manifestations of acute toxoplasmosis are partially mediated by an overproduction of the patient’s pro-inflammatory cytokines, e.g. tumor necrosis factor alpha (TNF-α), interleukin-1 (IL-1) and interferon gamma (IFN-γ) [6], all of which are important in limiting the parasite’s growth [7–9].

There is growing interest in exploring the link between infection with this parasite and autoimmune diseases, given the propensity of *T. gondii* infection to occur in immunocompromised patients [10–14]. Opportunistic infection with *T. gondii* is an increasing problem in association with inflammatory rheumatoid arthritis (RA) [15–17]. A heightened risk of *T. gondii* infection in patients with rheumatic diseases can be attributed to alterations in innate and adaptive immune responses [18]. Patients with RA were found to be highly susceptible to *T. gondii* infection - particularly during periods of immunosuppression that followed treatment with TNF-α inhibitors [19]. Contrasting data, however, suggest that *T. gondii* infection may ameriolate the severity of arthritis - delaying its onset in IL-1 receptor antagonist-deficient mice via *T. gondii*-derived Th1 immune response against Th17 cell-mediated arthritis [20].

Any reduction in the body’s defences against infection places arthritis patients at risk. Recognising the early symptoms of infection, while knowing the factors that increase susceptibility to *T. gondii* infection in patients with arthritis, will enable medical professionals to better assess patients’ needs, plan preventative therapy and initiate supportive measures. Several questions remain unanswered: Firstly, it is not clear whether there is an association between *T. gondii* infection and an increased risk of all forms of arthritis, or if the risk is limited to specific forms of arthritis. Secondly, it is unclear whether demographic or lifestyle variables increase the risk of *T. gondii* infection in arthritis patients. Thirdly, the prevalence of *T. gondii* infection in arthritis patients in China is still unknown. The present study was designed to investigate any possible association between *T. gondii* infection and arthritis by assessing the seroprevalence of, and risk factors associated with, *T. gondii* infection in patients with defined clinical forms of arthritis in eastern China.

## Methods

### Study sites

**Fig. 1:**
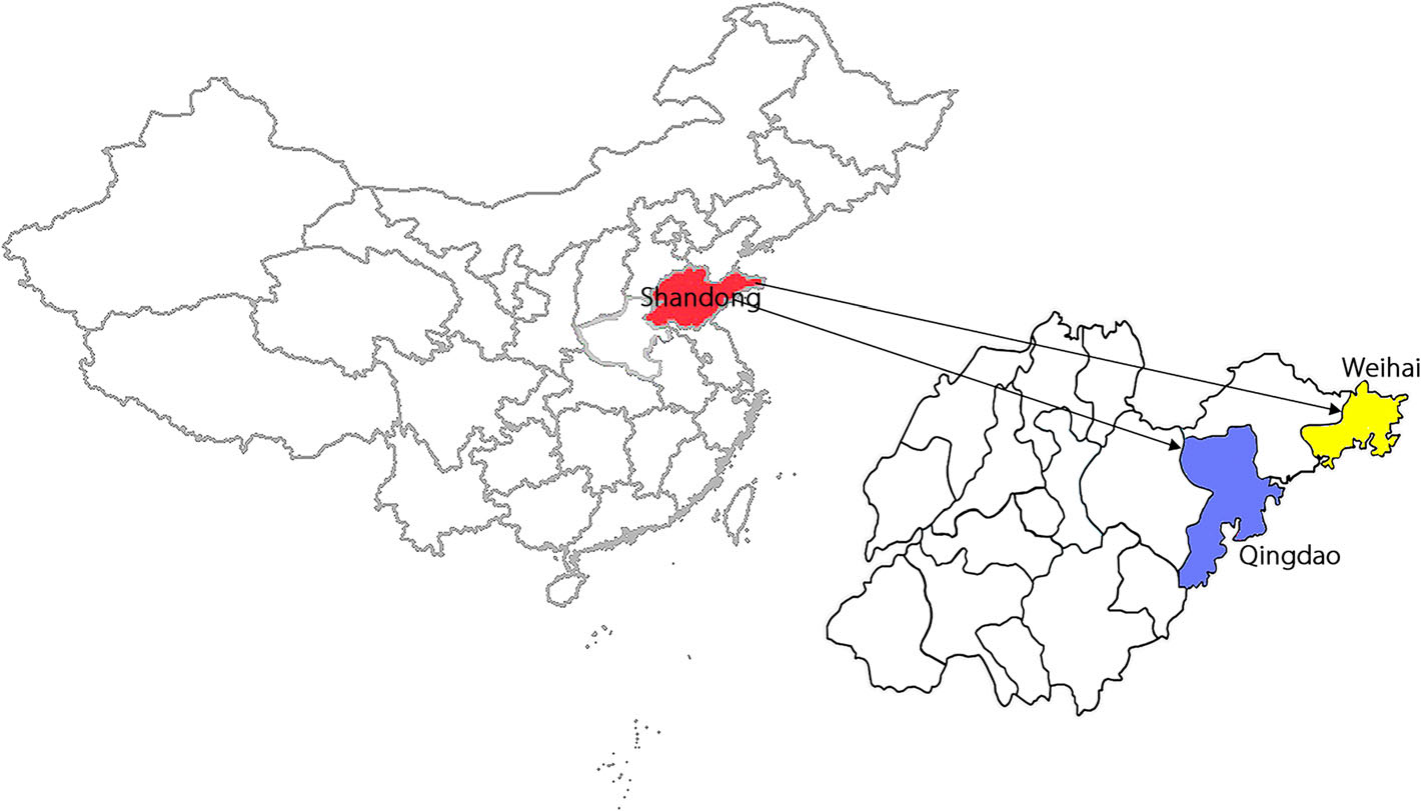
Maps of the study sites showing the location of Qingdao and Weihai cities in Shandong province, eastern China

The study was conducted in two cities, Qingdao and Weihai, in Shandong Province, eastern China (Fig. 1). Qingdao is located at the south-eastern tip of Shandong Province (35°35′ – 37°09′N, 119°30′ – 121°00′E) and Weihai is located at the eastern tip of Shandong province (36°41′ – 37°35′N, 121°11′ – 122°42′E).

### Study design and data collection

A case-control study was conducted in order to identify the prevalence of, and risk factors associated with, *T. gondii* seropositivity in patients with arthritis. The study was undertaken between December 2014 and November 2015, and included 820 inpatients hospitalized with the diagnosis or for treatment of arthritis. The clinical cases included patients with rheumatoid arthritis (*n* = 238), reactive arthritis (*n* = 122), osteoarthritis (*n* = 142), infectious arthritis (*n* = 109) and gouty arthritis (*n* = 209). An equivalent number of healthy control subjects (*n* = 820), of similar age and gender and from the same geographic region, were included in the study. We included only those individuals who had no history or current evidence of infection or comorbidities. Information on demographics; such as age, gender and area of residence, was obtained from the computerized inpatient registry of cases, or by asking the control individuals. A questionnaire was distributed to adult individuals, and to the parents/guardians of children, in order to obtain information about their lifestyle and feeding habits - including any history of contact with cats in the home (Yes/No), contact with pigs (Yes/No), the consumption of raw vegetables (Yes/No), consumption of raw/undercooked meat (Yes/No) and exposure to soil (Yes/No). All personal information was anonymized and treated as strictly confidential.

### Serum collection and serological testing

Approximately 5 ml of venous blood was drawn from each participant. Blood samples were stored overnight at ambient temperature, in order to allow blood clot formation, and then centrifuged at 1000×*g* for 10 min. The sera were collected in 2-ml Eppendorf tubes and kept at 4 °C for 24 – 48 h, until they were transported in an icebox to the State Key Laboratory of Veterinary Etiological Biology, Lanzhou Veterinary Research Institute, Chinese Academy of Agricultural Sciences, Gansu Province. Samples were stored (−20 °C) at the State Key Labs until analysis. Serum samples were analyzed for the presence of anti-*T. gondii* IgG and IgM antibodies using commercially available ELISA kits (Demeditec Diagnostics GmbH, Germany), according to the manufacturer’s instructions. Positive and negative serum controls were included in each plate. There was a concern that sera from RA patients might contain Rheumatoid factor (RF) autoantibody, which could potentially cross react non-specifically with IgM, leading to a false-positive result in the *T. gondii* IgM ELISA test [21]. Future work should consider using RF Neutralization Reagents (RFNR) to remove the RF from positive IgM specimens, then retesting these specimens. Samples would not be interpreted as positive for the specific IgM antibodies, unless they remained positive following adsorption with the RFNR.

### Statistical analysis

Statistical analysis was performed using the statistical software SPSS v19.0. For the univariate analysis, Chi-square test or Fisher’s exact test provided a comparison of the categorical variables. The Mantel-Haenszel test was used to probe any differences between patient and control groups. Multivariate logistic regression models were used to adjust for potential confounders. Variables associated with *T. gondii* infection in univariate analysis (*P* ≤ 0.10) were included in a multivariate logistic regression analysis. Odds ratios (*OR*s) and the corresponding 95% confidence interval (*CI*) were calculated, in order to identify independent risk factors for *T. gondii* infection. Results with a *P*-value <0.05 were considered as statistically significant.

## Results

### Epidemiology of arthritic patients with *T. gondii* infection

A total of 1640 individuals (820 arthritis patients and 820 health controls) were examined between December 2014 to November 2015. A significant difference (*P* < 0.001) was detected in the level of anti-*T. gondii* IgG antibodies in 154 arthritis patients (18.8%) vs. 98 control subjects (12%). Twelve patients tested positive for *T. gondii* IgM antibodies (prevalence, 1.5%), compared with 10 controls (1.2%) (*P* = 0.66). The details of arthritis patients and control subjects, including age distribution, gender, geographic region and area of residence are shown in Table 1. There was no evidence of a statistically significant association between being *T. gondii* seropositive and any of the tested variables, except for the ≤30-year-old age group (26.5%) and the >70-year-old age group (28.9%), which recorded the highest prevalence (Table 1). Our analysis revealed no significant differences between female (21.2%) and male (18.8%) arthritis patients (*P* = 0.414). *T. gondii* infection seroprevalence was not significantly different between urban and rural areas (17.9% vs. 22.2%; *P* = 0.128).

**Table 1:** Socio-demographic characteristics of the study population and seroprevalence of *Toxoplasma gondii* infection

Variables	Arthritis patients (*n* = 820)			Control subjects (*n* = 820)			Patients vs controls P* value
	Sero-prevalence of *T. gondii* infection			Sero-prevalence of *T. gondii* infection			
	No. tested	No. positive (%)	P* value	No. tested	No. positive (%)	P* value	
Age group (years)							
≤ 30	102	27 (26.5)	Reference	114	18 (15.8)	Reference	0.054
31 – 40	154	32 (20.8)	0.290	138	20 (14.5)	0.775	0.161
41 – 50	178	44 (24.7)	0.746	166	23 (13.9)	0.653	0.011
51 – 60	189	30 (15.9)	0.030	220	26 (11.8)	0.309	0.234
61 – 70	145	18 (12.4)	0.005	133	15 (11.3)	0.299	0.770
>70	52	15 (28.9)	0.754	49	5 (10.2)	0.348	0.019
Gender							
Male	314	59 (18.8)	Reference	366	41 (11.2)	Reference	0.005
Female	506	107 (21.2)	0.414	454	66 (14.5)	0.159	0.008
Geographic region							
Qingdao	393	77 (19.6)	Reference	380	53 (14.0)	Reference	0.036
Weihai	427	89 (20.8)	0.656	440	54 (12.3)	0.478	0.001
Residence area							
Urban	379	68 (17.9)	Reference	387	44 (11.4)	Reference	0.036
Rural	441	98 (22.2)	0.128	433	63 (14.6)	0.177	0.001

*Estimated using the *χ* ^2^ test

### Seropositivity among patients with arthritis

Seroprevalence is presented in Table 2, by each clinical form of arthritis. The highest seropositivity rate of *T. gondii* infection was detected in patients with rheumatoid arthritis (24.8%), followed by reactive arthritis (23.8%), osteoarthritis (19%), infectious arthritis (18.4%) and gouty arthritis (14.8%). Patients with rheumatoid arthritis and reactive arthritis had significantly higher seroprevalence when compared with control subjects (*P* < 0.001 and *P* < 0.002, respectively).

**Table 2:** Clinical diagnosis and seroprevalence of *Toxoplasma gondii* in arthritis patients in eastern China

Clinical diagnosis	Patients with anti-*T. gondii* antibodies				
	No. tested	No. positive	% (95% *CI*)	*OR* (95% *CI*)	*P* value
Rheumatoid arthritis	238	59	24.8 (19.48 – 30.70)	2.20 (1.53 – 3.14)	<0.001
Reactive arthritis	122	29	23.8 (16.05 – 30.84)	2.09 (1.31 – 3.30)	0.002
Osteoarthritis	142	27	19.0 (13.38 – 25.90)	1.56 (0.98 – 2.49)	0.060
Infectious arthritis	109	20	18.4 (10.89 – 26.26)	1.50 (0.89 – 2.53)	0.132
Gouty arthritis	209	31	14.8 (10.05 – 19.71)	1.16 (0.75 – 1.79)	0.500
Total	820	166	20.2 (17.44 – 23.01)	1.69 (1.30 – 2.21)	<0.001

### Domestic risk factors associated with *T. gondii* infection

Univariate analysis showed some lifestyle variables with a *P* value ≤0.25, including: contact with cats in the home, contact with pigs, the consumption of raw vegetables or raw/undercooked meat, and exposure to soil. In our multivariate analysis, only contact with cats in the home (*OR*, 1.68; 95% *CI*, 1.24 to 2.28; *P* = 0.001) was associated with significantly increased odds of *T. gondii* infection in arthritis patients (Table 3). There was no evidence of a significant association between patients’ *T. gondii* status and the other variables (i.e., contact with pigs, consumption of raw vegetables or raw/undercooked meat, or exposure to soil).

**Table 3:** Multivariate analysis of selected variables of arthritis patients and their association with *Toxoplasma gondii* infection

Variables	Adjusted odds ratio (*OR*)	95% Confidence interval (*CI*)	*P* value
Contact with cats at home	1.68	1.24 – 2.28	0.001
Contact with pigs	1.25	0.90 – 1.72	0.184
Consumption of raw vegetables	0.96	0.70 – 1.31	0.778
Consumption of raw/undercooked meat	1.11	0.93 – 1.33	0.247
Exposure with soil	0.84	0.62 – 1.14	0.266

## Discussion

*Toxoplasma gondii* infection is increasingly being reported in arthritis patients [15–17]; however, both the epidemiology and impact of this have been unclear. We assessed the risk of *T. gondii* infection among 820 arthritis patients from Shandong province, eastern China, during the period from December 2014 to November 2015; and determined the seroprevalence of anti-*T. gondii* antibodies in arthritis patients versus an equal number of healthy controls. Patients with arthritis were more likely to be infected with *T. gondii* (18.8% compared to 12% of healthy controls, *P* < 0.001), based on IgG seropositivity. Correlation between *T. gondii* infection and RA has been reported in other geographical areas, for example Iraq (54.0%) [22], Tunisia (58.4%) [23], Europe (63.0%) [17], and Egypt (54.0% [24] and 76.7% [25]). The higher seroprevalence of anti-*T. gondii* IgG antibodies among RA patients versus control patients reflects an association between latent *T. gondii* infection and RA. Contrastingly, there was no signficant difference between RA patients and controls with regard to the level of IgM antibodies, which was found to be consistent with a previous study reporting no anti-*T. gondii* IgM in RA patients [25]. Among the various clinical forms of arthritis, seroprevalence rates of only rheumatoid arthritis (24.8%; *P* < 0.001) and reactive arthritis (23.8%; *P* = 0.002) were significantly raised when compared with controls.

An increased risk of *T. gondii* infection in RA patients may be anticipated due to the disease-related immunological changes that compromise adaptive cellular immunity - crucial for the control of an intracellular pathogen such as *T. gondii*. RA is associated with alterations in the T cell repertoire [26], a reduction in clonal expansion of naïve T cells in response to a previously unknown antigen [27] and a reduction in newly generated naïve T cells migrating from the thymus into the periphery [27]. The lack of clinical information on the treatment history of study participants has precluded establishing corroborative evidence for any link between the use of immunosuppressive treatment and the frequency of *T. gondii* seropositivity in RA patients. Immunosuppressive therapies frequently used in the treatment of RA (especially TNF-α inhibitors) have been known to induce reactivation of latent *T. gondii* infection in arthritis patients, and may increase their propensity to acquire new opportunistic infections such as *T. gondii* or tuberculosis [28]. Clinical cases of cerebral toxoplasmosis and chorioretinitis have been reported in RA patients undergoing treatment with TNF-α antagonists [29–32]. Given the important role of TNF-α in controlling the growth of *T. gondii*, infection with opportunistic pathogens such as *T. gondii* can be a major safety concern in patients receiving this therapy. Measures should therefore be taken to prevent such opportunistic infections.

Physicians who prescribe TNF-α inhibitor drugs must understand the therapeutic implications on patients’ immunity and the potential risks of infection, in order to maximize therapeutic benefits and minimize adverse effects. In rheumatology practice, it is common practice to screen for tuberculosis, HBV, HCV, HIV and *varicella zoster* virus antibodies prior to the initiation of anti-TNF-α therapy in patients. Patients with arthritis should be monitored for early signs of opportunistic infection and, if confirmed, it may be necessary to withdraw anti-TNF-α therapy until the infection is properly treated. One study, based on a small sample of only 30 individuals, reported no difference in the level of anti-*T. gondii* IgG antibodies among RA patients undergoing treatment with traditional disease-modifying antirheumatic drugs or biological TNF inhibitors [25].

Our results showed the highest seroprevalence in individuals within the ≤30-year-old age and >70-year-old age groups. Young and elderly individuals may have less efficient immunity to control opportunistic infections, or be more likely to interact with cats than those within other age groups. Previous studies have also shown an association between age and seropositivity of *T. gondii* [5, 33–35]; with higher *T. gondii* seroprevalence reported in older RA patients [17]. In our study, no significant difference in the seropositive rates was observed between males and females (*P* = 0.414). However, we detected a significant association between contact with cats in the home and *T. gondii* seropositivity (*OR*, 1.68; 95% *CI*, 1.24 to 2.28; *P* = 0.001), contributing to the body of evidence suggesting that contact with cats is associated with an increased risk of *T. gondii* infection [5, 35, 36]. Felines are the primary host of *T. gondii*, whose oocytes pass with the animal’s stool and cause toxoplasmosis if ingested by humans or any other intermediate host. Although cats are popular as domestic pets in China, little attention has been paid to their role in environmental contamination with *T. gondii* oocysts [37, 38]. It is important to inform the general public and medical professionals about the risk factors of *T. gondii* infection - in particular the important role cats can play in transmitting *T. gondii* infection to arthritis patients.

Although the association between contact with pigs and *T. gondii* seropositivity was not statistically significant (*P* = 0.184), it deserves some attention. The identification of contact with cats and with pigs as the two most important risk factors, is of interest and to be expected. Human infection can occur via cat-to-pig and pig-to-human transmission [39]. *T. gondii* will always be a risk while cats are maintained in the swine environment; cats shed millions of oocysts which can survive in the environment for months or even years, with a single oocyst having the potential to cause full-blown infection in a pig. Pork has been implicated as the meat most commonly associated with food-borne toxoplasmosis [5]. People who handle pigs, or consume food within pig facilities, may be less likely to wash their hands before eating or after handling raw meat, leaving them at higher risk of infection [40, 41].

## Conclusions

This study has demonstrated a high correlation between anti-*T. gondii* antibodies and arthritis for the first time in a population from eastern China, suggesting an increased susceptibility to opportunistic *T. gondii* infection in arthritic patients. It remains to be elucidated if chronic *T. gondii* infection was a trigger for the development of arthritis; whether disease-related immunosuppression promoted the reactivation of a latent infection; or if arthritis patients were idopathically predisposed to novel infections. TNF-α inhibitors used for the treatment of rheumatic diseases can increase the risk of frank toxoplasmosis; therefore, screening for latent *T. gondii* infection is strongly recommended before initiating such therapy. Our study adds to the body of evidence that contact with cats is associated with an increased risk of *T. gondii* infection. These findings should inform Public Health policy on the risk of opportunistic infections in arthritis patients.

## Additonal file

Additional file 1: Multilingual abstracts in the five official working languages of the United Nations. (PDF 389 kb)

## Abbreviations

CI: Confidence interval
ELISA: Enzyme-linked immunosorbent assay
HBV: Hepatitis B virus
HCV: Hepatitis C virus
HIV: Human immunodeficiency virus
IFN-γ: Interferon gamma
IgG: Immunoglobulin G
IgM: Immunoglobulin M
IL-1: Interleukin-1
OR: Odds ratio
SPSS: Statistical Packages for Social Sciences
TNF-α: Tumor necrosis factor-alpha
