# Supplementary material for: Seroprevalence of Toxoplasma gondii infection in arthritis patients in eastern China
Source: Infect Dis Poverty. 2017 Oct 25;6:153. doi: 10.1186/s40249-017-0367-2 (PMC5655948; doi:10.1186/s40249-017-0367-2)
Supplement: Additional file 1: — Multilingual abstracts in the five official working languages of the United Nations. (PDF 389 kb) [file 40249_2017_367_MOESM1_ESM.pdf]

Translation of the abstract into the five official working languages of the United Nations

معدل الانتشار المصلي للإصابة بالمقوسات الغوندية لدى مرضى التهاب المفاصل في شرقي الصين

آي-لينغ تيان، يوان-لين غو، نا تشو ، وي تسونغ، غوانغ-شينغ لي، هاني م. الشيخة وشينغ- تشوان تشو

ملخص

خلفية: هناك أدلة متراكمة عن زيادة القابلية للتعرض للإصابة لدى المرضى الذين يعانون من التهاب المفاصل. سعينا إلى فهم وبائيات عدوى المقوسات الغوندية لدى مرضى التهاب المفاصل في شرقي الصين، نظراً لندرة البيانات عن حجم الإصابة بالمقوسات الغوندية لدى هؤلاء المرضى.

الطرائق: جرى تقييم عدوى الانتشار المصلي للمقوسات الغوندية من خلال فحص مُمْتَزٌ مَناعِيّ مرتبط انزيمياً باستخدام مستضد طفيلي خام على 820 مريض بالتهاب المفاصل وعلى عدد متساو من الضوابط الصحية من مدينتي تشينغداو و ويهاي شرقي الصين. وجرى أيضاً الحصول على معلومات اجتماعية ديموغرافية وسريرية وعن نمط حياةالمشاركين بالدراسة

نتائج: كان انتشار مضاد IgGللمقوسات الغوندية أعلى بشكبٍ ذي دلالة لدى مرضى التهاب المفاصل (بنسبة 18.8%) مقارنة مع 12% في ضوابط صحية (قيمة الاحتمالية < 0.001). وقد وجد أن اثنا عشر شخصٍ من مرضى التهاب المفاصل لديهم مضاداتالمقوسات الغوندية من نوع الأجسام المضادة IgM – مقارنة مع 10 من مرضى المراقبة (1.5% مقابل 1.2%). لم تؤثر العوامل الديموغرافية بشكلٍ كبير على هذه الترددات للانتشار المصلي. تم اكتشاف أعلى معدل للإصابة بالمقوسات الغوندية لدى المرضى الذين يعانون من التهاب المفاصل الروماتويدي (24.8 %)، تليها التهاب المفاصل الارتكاسي (23.8 %) وهشاشة العظام (19 %) والتهاب المفاصل المُعدي (18.4 %) والتهاب المفاصل النقرسي (14.8 %). وكانت معدلات الانتشار المصلي لالتهاب المفاصل الروماتويدي والتهاب المفاصل الارتكاسي أعلى بكثير بالمقارنة مع عناصر التحكم (قيمية الاحتمالية < 0.00 و قيمة الاحتمالية < 0.05، على التوالي). تم الكشف عن وجود ارتباط كبير بين العدوى بالمقوسات الغوندية ووجود القطط في منازل مرضى التهاب المفاصل (نسبة الرجحان [*أو*]، 1.68؛ فاصل الثقة 95 ٪ [*فاصل الثقة*]: 1.24 – 2.28؛ *قيمة الاحتمالية* = 0.001).

النتائج:<bx/>تنسجم هذه النتائج مع النتائج السابقة وتوسعها موفرةً المزيد من الأدلة لدعم وجود صلة بين الاتصال مع القطط وزيادة خطر العدوبالمقوسات الغوندية ودراستنا أيضاً هي أول دراسة تؤكد الارتباط بين الإصابة بالمقوسات الغوندية مع مرضى االتهاب المفاصل في الصين. ويجري مناقشة الآثار المترتبة على وقاية أفضل وسيطرة على العدوى بالمقوسات الغوندية لدى مرضى التهاب المفاصل.

Translated from English version into Arabic by Abd Al-Rahman Al-Midani, through

TRANSLATORS
WITHOUT BORDERS

中国东部地区关节炎患者弓形虫感染的血清学调查

田艾灵，谷源林，周娜，丛伟，栗广兴，Hany M. Elsheikha，朱兴全

摘要

引言：越来越多的证据表明，关节炎患者更易感染其它疾病。然而关于关节炎患者的弓形虫感染情况的报道很少，为此我们进行了中国东部地区关节炎患者弓形虫感染的血清流行病学调查。

方法：我们选取了生活在中国山东省青岛市和威海市的 820 例关节炎患者以及同等数量的

健康对照，用酶联免疫吸附试验（ELISA）检测其弓形虫感染的血清阳性率。同时对参与者的社会人口特征、临床特征和生活方式也进行了调查。
结果：我们发现，关节炎患者中抗弓形虫 IgG 抗体的阳性率为 18.8%，健康对照组的阳性率为 12%，前者的感染率显著高于后者（$P < 0.001$）；关节炎患者中抗弓形虫 IgM 抗体的阳性率为 1.5%（12/820），健康对照组的阳性率为 1.2%（10/820）。而在本次试验中，社会人口特征对关节炎患者的弓形虫血清阳性率并没有显著的影响。在对关节炎患者的临床特征调查中发现，类风湿关节炎患者的弓形虫感染率最高（24.8%），其次为反应性关节炎患者（23.8%），骨性关节炎患者（19%），感染性关节炎患者（18.4%）和痛风性关节炎患者（14.8%）。类风湿关节炎患者弓形虫感染率和反应性关节炎患者的弓形虫感染率分别显著高于其健康对照组（$P < 0.001$，$P < 0.05$）。多变量分析中显示，家中养猫是关节炎患者感染弓形虫的唯一危险因素（*OR*, 1.68; 95% *CI*: 1.24 – 2.28; $P = 0.001$）。
结论：本研究结果与之前的发现一致，并提供了进一步的证据表明，与猫的接触能够增加弓形虫感染的风险率。我们的研究首次证实中国关节炎患者与弓形虫感染存在相关性。研究结果提示我们，应重视关节炎患者弓形虫感染的预防和控制。

Translated from English version into Chinese by Ai-Ling Tian, edited by Xing-Quan Zhu

Séroprévalence de l'infection par *Toxoplasma gondii* chez les patients atteints d'arthrite dans l'est de la Chine

Ai-Ling Tian, Yuan-Lin Gu, Na Zhou, Wei Cong, Guang-Xing Li, Hany M. Elsheikha et Xing-Quan Zhu

Résumé
Contexte : Il y a de plus en plus de preuves qui démontrent une sensibilité accrue aux infections chez les patients souffrant d’arthrite. Nous avons cherché à comprendre l’épidémiologie de l’infection par *Toxoplasma gondii* chez les patients atteints d'arthrite dans l'est de la Chine, compte tenu de la rareté des données sur l’ampleur de l’infection par *T. gondii* chez ces patients.
Méthodes : La séroprévalence de l'infection par *T. gondii* a été évaluée par des essais d'immuno-absorption enzymatique, en utilisant un antigène brut du parasite chez 820 patients arthritiques et un nombre égal de sujets en bonne santé, du , Qingdao et des villes de Weihai, Chine orientale. Des renseignements socio-démographiques, médicaux et sur les habitudes de vie des participants ont également été obtenus.
Résultats : La prévalence d'anticorps IgG anti*T. gondii* était significativement plus élevée chez les patients arthritiques (18,8 %), comparé à 12 % chez les sujets en bonne santé ($P < 0,001$). Douze patients souffrant d’arthrite avaient des anticorps IgM anti*T. gondii* – comparables à 10 patients des sujets en bonne santé (1,5 % *contre* 1,2 %). Les facteurs démographiques n'ont pas eu d'influence importante sur ces fréquences de séroprévalence. Le taux le plus haut de séropositivité à l'infection par *T. gondii* a été détecté chez des patients atteints de polyarthrite rhumatoïde (24,8 %), suivi de l’arthrite réactionnelle (23,8 %), l’arthrose (19 %), l’arthrite septique (18,4 %) et l’arthrite goutteuse (14,8 %). La séroprévalence de la polyarthrite rhumatoïde et de l’arthrite réactionnelle était considérablement plus élevée par rapport, respectivement, aux sujets en bonne santé ($P < 0,00$ et $P < 0,05$). Une association significative a été observée entre l’infection par *T. gondii* et les chats

présents au domicile chez les patients arthritiques (rapport des cotes [*OR*], 1,68 ; intervalle de confiance 95 % [*CI*] : 1,24 – 2.28 ; *P* = 0,001).

Conclusions : Ces résultats sont conformes et étendent les résultats déjà obtenus, apportant des preuves additionnelles en appui au lien entre le contact avec les chats et un risque accru d'infection par *T. gondii* </bx>. Notre étude est aussi la première à confirmer une association entre l'infection par *T. gondii* et les patients arthritiques en Chine. Les implications pour une meilleure prévention et un meilleur contrôle de l'infection par *T. gondii* chez les patients atteints d'arthrite sont abordées.

Translated from English version into French by Sabyna Delperdange, through

TRANSLATORS WITHOUT BORDERS

Серопревалентность инфекции *Toxoplasma gondii* у пациентов с артритом в Восточном Китае

Ай Лин Тянь, Юань Линь ГУ, На Чжоу, Вэй Цонг, Гуан Син Ли, Хэни М. Элшейха и Син Чуань Чжу

Аннотация

Контекст исследования: Появляется все больше доказательств повышенной восприимчивости пациентов с артритом к данной инфекции. Мы пытались понять эпидемиологию инфекции *Toxoplasma gondii* у больных артритом в восточной части Китая, учитывая нехватку данных о масштабах инфекции *T. gondii* у данных пациентов.

Методы: Уровень серопревалентности инфекции *T. gondii* был оценен с помощью иммуноферментного анализа посредством введения неочищенного антигена вируса в организм 820 людей, страдающих артритом, и соответствующему количеству здоровых представителей контрольной группы из городов Циндао и Вейхай, Восточный Китай. Были также изучены социо-демографические, клинические данные, информация об образе жизни участников исследования.

Результаты: Показатель распространенности антител класса IgG к вирусу*T. gondii* был значительно выше у пациентов с артритом (18,8%), чем у здоровых пациентов контрольной группы (12% (*П.* <0,001). У двенадцати пациентов с артритом были обнаружены антитела класса IgM к вирусу*T. gondii*, что сопоставимо с 10 пациентами контрольной группы (1,5% *по сравнению* 1,2%). Демографические факторы существенно не влияют на уровень серопревалентности. Самый высокий показатель серопозитивной реакции на инфекцию *T. gondii* был обнаружен у больных с ревматоидным артритом (24,8%), затем с реактивным артритом (23,8%), артрозом (19%), инфекционным артритом (18,4%) и подагрическим артритом (14,8%). Показатели серопревалентности ревматоидного артрита и реактивного артрита были значительно выше по сравнению с показателями здоровых пациентов контрольной группы (*П.* < 0.00 и *П.* < 0,05 соответственно). Была обнаружена существенная связь между инфекцией *T. gondii* и наличием в доме кошек у пациентов с артритом

(соотношение шансов [*или*], 1,68; 95% доверительный интервал [*CI*]: 1,24 – 2,28; *П.* = 0,001).
Заключение: Данные выводы дополняют и согласуются с предыдущими результатами, предоставляя дополнительные доказательства того, что существует связь между контактом с кошками и повышенным риском инфекции *T. gondii*. В нашем исследовании также впервые подтверждается взаимосвязь между инфекцией *T. gondii* и больных артритом в Китае. Меры по предупреждению и борьбе с инфекцией *T. gondii* у больных артритом, находятся на стадии обсуждения.

Translated from English version into Russian by Karina1207, through

Seroprevalencia de la infección *Toxoplasma gondii* en pacientes de artritis en el este de China

Ai-Ling Tian, Yuan-Lin Gu, Na Zhou, Wei Cong, Guang-Xing Li, Hany M. Elsheikha y Xing-Quan Zhu

Resumen

Antecedentes: Existen cada vez más evidencias de una mayor susceptibilidad a la infección en pacientes con artritis. Buscamos entender la epidemiología de la infección por *Toxoplasma gondii* en los pacientes con artritis en el este de China, dada la escasez de datos acerca de la magnitud de la infección por *T. gondii* en estos pacientes.
Métodos: La seroprevalencia de la infección por *T. gondii* se calculó por medio de un ensayo inmunoabsorbente ligado a enzimas usando un antígeno del parásito en 820 pacientes con artritis y una cantidad igual de controles sanos en las ciudades de Qingdao y Weihai, al este de China. También se obtuvo información sociodemográfica y del estilo de vida de los participantes del estudio.
Resultados: La prevalencia de anticuerpos IgG contra el parásito *T. gondii* fue significativamente más alta (18.8%) en los pacientes con artritis en comparación con el 12% obtenido en los controles saludables ($P< 0.001$). Doce pacientes con artritis presentaron anticuerpos IgM contra el *T. gondii*, mientras que en el grupo de pacientes de control fueron 10 (1.5% *contra* 1.2%). Los factores demográficos no influyeron significativamente en estas frecuencias de seroprevalencia. La tasa más alta de seropositividad de la infección por *T. gondii* se detectó en pacientes con artritis reumatoide (24.8%), seguida por la artritis reactiva (23.8%), la osteoartritis (19%), la artritis infecciosa (18.4%) y la artritis gotosa (14.8%). Las tasas de seroprevalencia de la artritis reumatoide y la artritis reactiva es fueron significativamente más altas al compararse con el grupo de control ($P< 0.00$ y $P< 0.05$, respectivamente). Se detectó una asociación significativa entre la infección por *T. gondii* y la presencia de gatos en los hogares de los pacientes con artritis (índice de probabilidad [*OR*], 1.68; 95% de intervalo de confianza [*CI*]: 1.24 – 2.28; $P = 0.001$).
Conclusiones: Estos hallazgos son consistentes con los resultados anteriores y los amplían, lo cual aporta más evidencias que relacionan el contacto con los gatos y un aumento del riesgo de infección

por *T. gondii*. Nuestro estudio es también el primero en confirmar una relación entre la infección por *T. gondii* y los pacientes con artritis en China. Se debaten las implicaciones para una mejor prevención y control de las infecciones por *T. gondii* en pacientes con artritis.

Translated from English version into Spanish by Carmen Garcia, through

TRANSLATORS WITHOUT BORDERS
